# Health Insurance Notes

**Published:** 1923-07

**Authors:** 


### NATIONAL HEALTH INSURANCE NOTES.

### THE SUCCESS OF PANEL DOCTORING.

It is somewhat unusual to come across such a heading as the above in the daily Press. The *Manchester Guardian*, in a recent issue, says, under such a headline, that medical men and the public alike are now generally satisfied that the panel system serves its purpose well; neither party would wish the system away. It is not pretended that the system has not its imperfections—it is, indeed, expressly stated in the same article that it has. But the statement made by so well-balanced and well-informed an organ of public opinion may be set in refreshing contrast to those of another daily paper which for a long time past has, with well-leaded headlines, been lashing itself—and very few other people, as far as we can judge—into a state of fury about the iniquities of the panel doctor. "Pretending to lash itself" would perhaps be nearer the truth.

### ARE PANEL DOCTORS OVER-DRIVEN?

It seems hardly worth while reiterating that some doctors scamp their work, and that the great majority do not. The proportions are about the same as in any other profession. And yet what are we to say, if not that, in answer to this type of criticism from the *Referee*, which says, "We make no attack on the panel doctors as a class. We simply point to the obvious truth that . . . . strive as hard as they may, they cannot give the time and the individual attention required for proper treatment." Now, the facts are that the majority of panel doctors have less than 1,000 insured persons on their lists, comparatively few have more than 2,000, and that the doctors are prepared to accept an absolute limitation of lists for any practitioner, working single-handed, to 2,500. If we take even this top figure—which applies to very few practices—it would be found that, of the 2,500 people, probably not more than 1,500 would need attention in any one year, and an average of four services would be found to meet their needs; $4 \times 1{,}500 = 6{,}000$ services in a year, which divided by 300 gives 20 services a day—a not inconsiderable number of which would be surgery attendances for trifling ailments. Is it seriously contended that this means an over-worked doctor, or that it does not leave him a considerable margin of time for private practice?

### LIMITATION OF LISTS.

It is the combination of insurance with private practice which makes the general limitation of panel lists, in the view of many doctors, a not undesirable thing, because it checks the possible growing-up of a class of purely insurance doctors—a class which, contrary to the popular impression (due to the attacks on "panel doctors") does not in fact at present exist. But it is the same combination of insurance with private practice which makes the application of a general limit a rather meaningless thing. The more effective application of a limit in individual cases where there are complaints of negligence would be much more useful, although admittedly more difficult.

### THE WEEDING-OUT PROCESS.

The Consultative Council to the Minister of Health are impresssed, we read, "by the difficulties that arise in the way of securing the effective consideration of certain cases of insurance doctors who are commonly believed to be habitually so behaving that their continuance on the list is prejudicial to the efficiency of the Service, and have asked the Ministry to confer with the representatives of the profession as to the possibility of removing these difficulties by some amendment of regulations or otherwise." We sympathise with the difficulties which the Consultative Council doubtless feel in themselves proposing any practical method of facilitating the removal of a doctor from the panel. Undoubtedly there are doctors who ought to be removed from the panel, against whom patients will not come forward as witnesses. But it cannot be overlooked that to remove a doctor from the panel is to deprive him of his livelihood, and he is therefore protected by the holding of a judicial enquiry. Where the Approved Societies, for whom, in the main, the Consultative Council speaks, are aware of cases of doctors really unfit to be on the panel, they must make the most thoroughgoing efforts to see that proper evidence is forthcoming; it is really the only way.

### INSULIN FOR PANEL PATIENTS.

Dr. Brackenbury points out in a letter to *The Times* that he can obtain any necessary supply of Insulin, free of cost, for his insured patients, whereas the professional man of small means must pay £180 a year for the drug alone or die. Meanwhile, he has to contribute towards the Insurance fund both as employer and taxpayer. We are not quite sure what is the moral of this, but it is instructive for those who have an idea that panel treatment consists of a hasty feeling of the pulse followed up by a bottle of coloured and sweetened water, or of a box of pills flung in the cottage doorway as the doctor dashes by in his Rolls-Royce.

### ADVERTISING ON THE MEDICAL CERTIFICATES.

We hope that the Minister of Health will deal faithfully with the Stationery Office next time they want to advertise butter and margarine on the back of a medical certificate. An advertisement for something more remote from health would have been less open to criticism, but the whole idea suggests rather a peddling form of raising revenue. The margarine advertisement has brought up a crop of questions in Parliament, with the result that, when Form 40B comes up next for revision, we doubt not that the advisers of the Minister will see to it that the advertisement is unavoidably crowded out for want of space.

